# Protein stability prediction by fine-tuning a protein language model on a mega-scale dataset

**DOI:** 10.1371/journal.pcbi.1012248

**Published:** 2024-07-22

**Authors:** Simon K. S. Chu, Kush Narang, Justin B. Siegel

**Affiliations:** 1 Biophysics Graduate Program, University of California Davis, Davis, California, United States of America; 2 College of Biological Sciences, University of California Davis, Davis, California, United States of America; 3 Genome Center, University of California Davis, Davis, California, United States of America; 4 Department of Chemistry, University of California Davis, Davis, California, United States of America; 5 Department of Biochemistry and Molecular Medicine, University of California Davis, Davis, California, United States of America; Universita degli Studi di Torino, ITALY

## Abstract

Protein stability plays a crucial role in a variety of applications, such as food processing, therapeutics, and the identification of pathogenic mutations. Engineering campaigns commonly seek to improve protein stability, and there is a strong interest in streamlining these processes to enable rapid optimization of highly stabilized proteins with fewer iterations. In this work, we explore utilizing a mega-scale dataset to develop a protein language model optimized for stability prediction. ESM_therm_ is trained on the folding stability of 528k natural and *de novo* sequences derived from 461 protein domains and can accommodate deletions, insertions, and multiple-point mutations. We show that a protein language model can be fine-tuned to predict folding stability. ESM_therm_ performs reasonably on small protein domains and generalizes to sequences distal from the training set. Lastly, we discuss our model’s limitations compared to other state-of-the-art methods in generalizing to larger protein scaffolds. Our results highlight the need for large-scale stability measurements on a diverse dataset that mirrors the distribution of sequence lengths commonly observed in nature.

## Author summary

Research in Professor Justin Siegel’s lab focuses on discovering and engineering enzyme catalysis. His work follows a design-build-test cycle, integrating computational protein modeling with wet-lab experiments. Key areas of his research include *de novo* enzyme design, enzyme therapeutics for celiac disease, and applications in food and renewable energy. Additionally, his lab has developed the Design2Data program, a multi-year, multi-campus effort to curate a high-quality dataset of enzymatic activity and stability for beta-glucosidase.

Under the supervision of Professor Justin Siegel, I am engaged in molecular modeling and machine learning in protein engineering. I have a background in molecular dynamics simulations for protein and cell membrane permeability estimation and in using the Rosetta molecular modeling suite for protein structure modeling and enzyme-substrate interaction. Current research topics include the prediction of mutational effects on protein functions and protein language models for functional prediction and protein design.

## Introduction

Protein stability is one of the foundations of protein engineering to design resilient proteins for industrial processes and therapeutic manufacturing [1–3]. Beyond protein engineering, destabilizing mutations are associated with pathogenicity, and stability predictors can help identify pathogenic mutations across human proteome [4–7]. Molecular modeling methods, including Rosetta [8, 9], FoldX [10], and molecular dynamics simulations [11], have been shown to predict the impact of mutation on protein stability. More recently, the use of machine learning models grounded in biophysical features and evolutionary statistics [12–20] has offered an alternative approach to stability and function prediction without the need for computationally intensive molecular modeling simulations. Fueled by the latest advances in deep learning, convolutional neural networks (CNNs) [21] and graph neural networks (GNNs) [22] are now being adopted to predict mutational impacts on stability by operating directly on the input protein structure [23, 24]. For example, RaSP is a CNN-based model trained on top of Rosetta [25], while ELASPIC-2, another stability predictor, operates on both sequence embedding from ESM and structural embedding from GNN [26–28].

Despite these advancements, the lack of a consistent and universal dataset remains an obstacle. While merging smaller datasets into a more comprehensive collection, such as ProTherm [29], ProtaBank [30] and ThermoMutDB [31], is a feasible approach, combined datasets often consist of closely related but distinct quantities accompanied by additional discrepancies in experimental conditions. While deep mutagenesis scanning (DMS) offers profound insights, these studies typically focus on a single protein target, limiting the broader applicability of the derived data and models subsequently trained on these datasets. In light of these challenges, Tsuboyama et al. introduced a mega-scale thermostability dataset, encompassing 776k short protein sequences derived from 479 small protein domains, all consistently evaluated using the same assay [32].

Utilizing this dataset, we fine-tuned a protein language model (pLM), named ESM_therm_, from ESM-2 [33] to act as an end-to-end stability predictor. We observe that ESM_therm_ performs comparably with state-of-the-art models and generalizes to small protein sequences distal to those of the training set. We also demonstrate that training on an ensemble of protein domains, instead of mutagenesis studies of a single domain, improves the performance of the fine-tuned protein language model for folding stability prediction. Lastly, we discuss the limitations of ESM_therm_ and compare it to other state-of-the-art methods in the ability to generalize to longer protein sequences.

## Results

### Evaluating model generalizability on test-set-only domains

**Fig 1:**
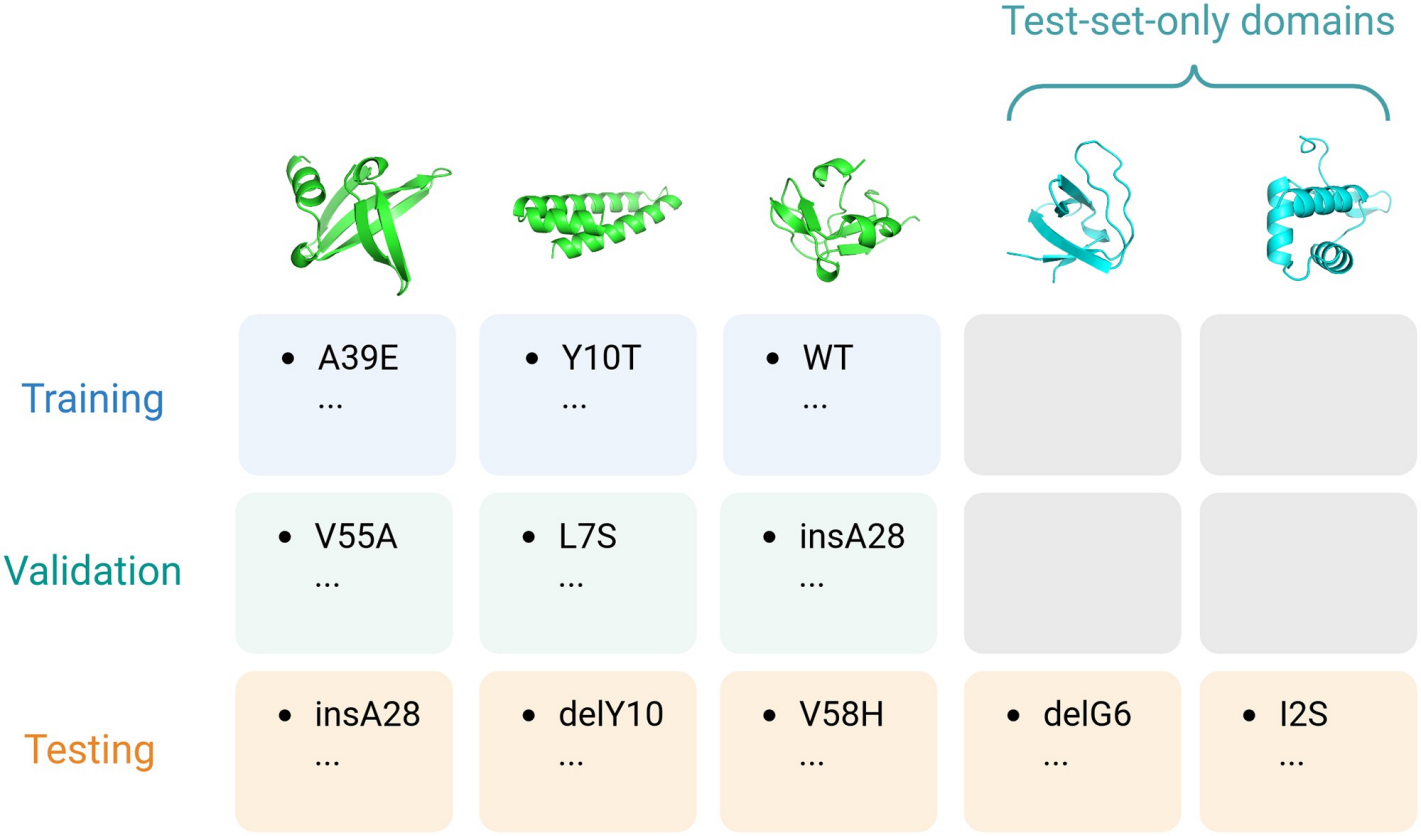
Dataset splitting scheme. Protein domains are first identified by their wildtype sequences and split into train-validation-test (green) and test-set-only partitions (cyan). Mutants are then randomly assigned to either training, validation and test sets or test set only according to their respective wildtype.

Protein stability prediction can be assessed on different scales of generalizability. Although machine learning algorithms are often trained and tested on different sets of non-overlapping samples, the definition of overlap is ambiguous in protein sequences. For example, assigning two point mutants from the same WW domain, one to the training set and another to the test set, can assess the generalizability of the model to sequences sharing the same protein domain. However, it fails to evaluate the generalizability of the model to a domain different from those in the training set, such as an SH2 domain. To benchmark our model on both scales, our test set sequences consist of two parts. The first part is formed by protein domains also found in training set, whereas the second part consists of protein domains exclusively found in test set only, denoted as test-set-only domains. We assess the model performance by Spearman’s R, and its capability to generalize to these test-set-only sequences by the highest sequence identity to any domains in the training set. Given that domains are classified according to the wildtype definitions by Tsuboyama et al. [32], it is possible for domains exclusive to the test set to still share considerable sequence identity with those in the training set. This setup allows for an assessment of generalizability across varying degrees of sequence identity. The dataset-splitting scheme is illustrated in Fig 1 and further detailed in Methods and Materials.

**Fig 2:**
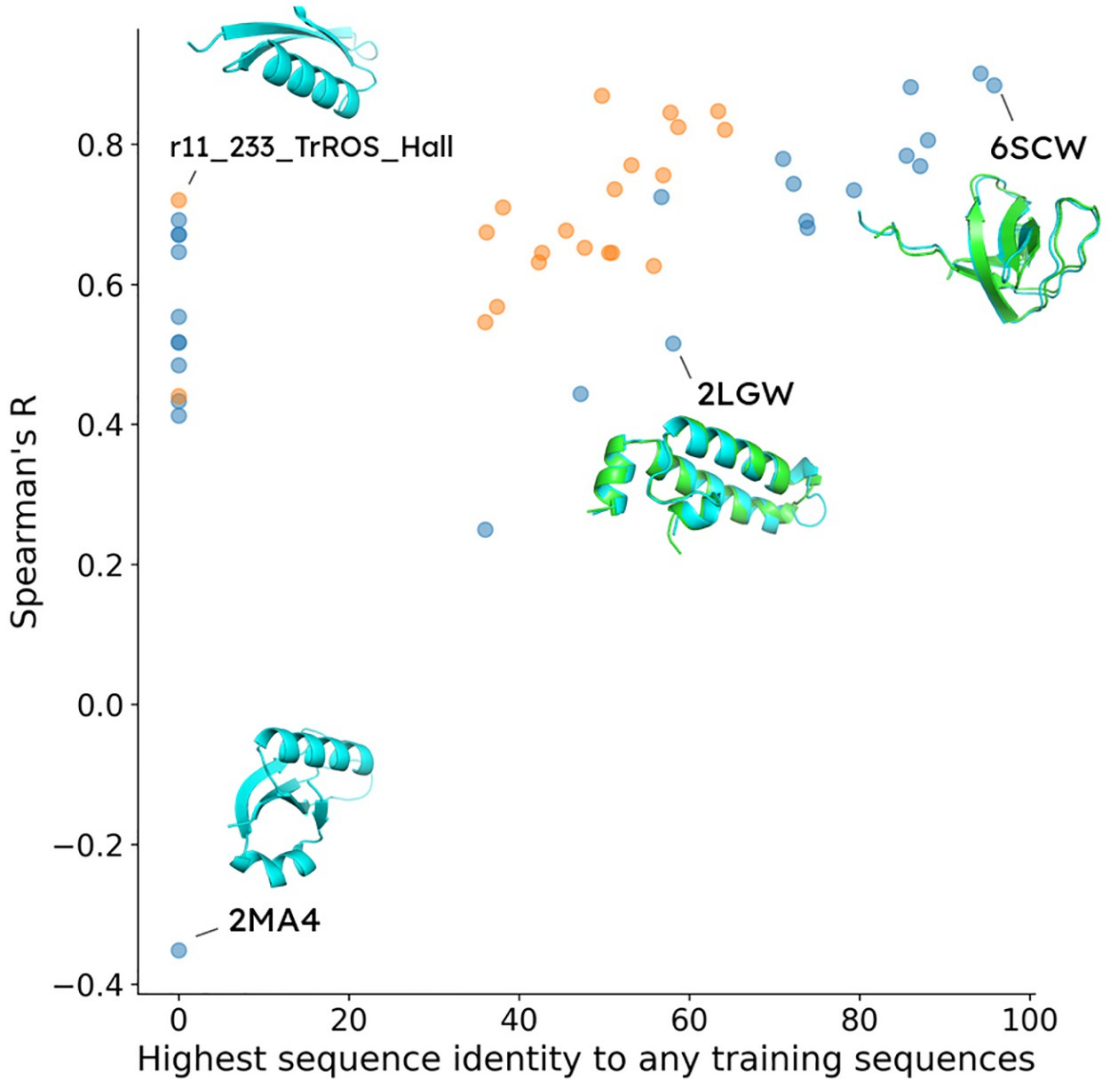
Spearman’s R on test-set-only protein domains. Natural protein domains are labeled in blue and *de novo* domains are in orange. The x-axis is the highest sequence identity from the evaluated protein domain to those in the training set. In the case where no sequence alignment was found, 0% is assigned. The y-axis is the Spearman’s R evaluated on all sequences from the corresponding domain. We highlighted some of the test-set-only AlphaFold2 models in cyan, and when possible, overlay them with the training-set protein domains of the highest sequence identity in green.

ESM_therm_ generalizes reasonably well to 47 test-set-only protein domains, illustrated in Fig 2. The Spearman’s R evaluated on individual domains ranges from 0.2 to 0.9, except for the uncharacterized bacterial protein yahO (PDB code: 2MA4) [34]. Among all test-set-only domains, SH3-subunit of chicken alpha spectrin (PDB code: 6SCW) [35] has the highest sequence identity of 95.8% and scores a corresponding Spearman’s R of 0.88. Going down the ladder to test-set-only domains in lower sequence identity, our model scores worse in Homo sapein J-domain protein HSJ1a (PDB code: 2LGW) [36] at 59% identity but still retains a Spearman’s R of 0.52.

In the 13 cases where no alignment with the training set sequences passes e-value < 10^−3^, ESM_therm_ is capable of generalizing to both natural and *de novo* proteins. No training sequence can be aligned to *Escherichia coli* DNA-binding arginine repressor (PDB code: 1AOY) [37], and yet its Spearman’s R evaluated is 0.69. For *de novo* designs, we highlight two protein domains from Baker Lab. αββα domain (HEEH_KT_rd6_0790) is a mini-protein from high-throughput computational design with Rosetta [38], whereas the trRosetta-hallucinated structure (r11_233_TrROS_Hall) was sampled with iterative sequence refinement to improve the confidence in the prediction of residue-residue distance map [39]. Spearman’s R on these domains is 0.44 and 0.72, respectively.

### Improving stability prediction by learning all domains collectively

Prior to the work by Tsuboyama et al. [32], DMS was often restricted to a single protein of interest. In the case where the target of interest is not thoroughly mapped, site-saturated mutagenesis studies from a homologous sequence(s) might provide insights into selecting the best mutation for the specific function of interest. However, direct cross-comparison between proteins is often complicated by the difference in measured quantities and experimental conditions between functional assays. This inconsistency makes it difficult to highlight the benefits of learning from multiple target proteins collectively in a systematic manner.

The mega-scale dataset addresses this difficulty by measuring folding stability across multiple protein domains in a uniform experimental condition, and it helps us compare two paradigms, i.e. transfer learning from homologous sequences and learning from all domains collectively. To contrast these approaches, we assess the generalizability of the model fine-tuned on these paradigms on test-set-only protein domains.

**Fig 3:**
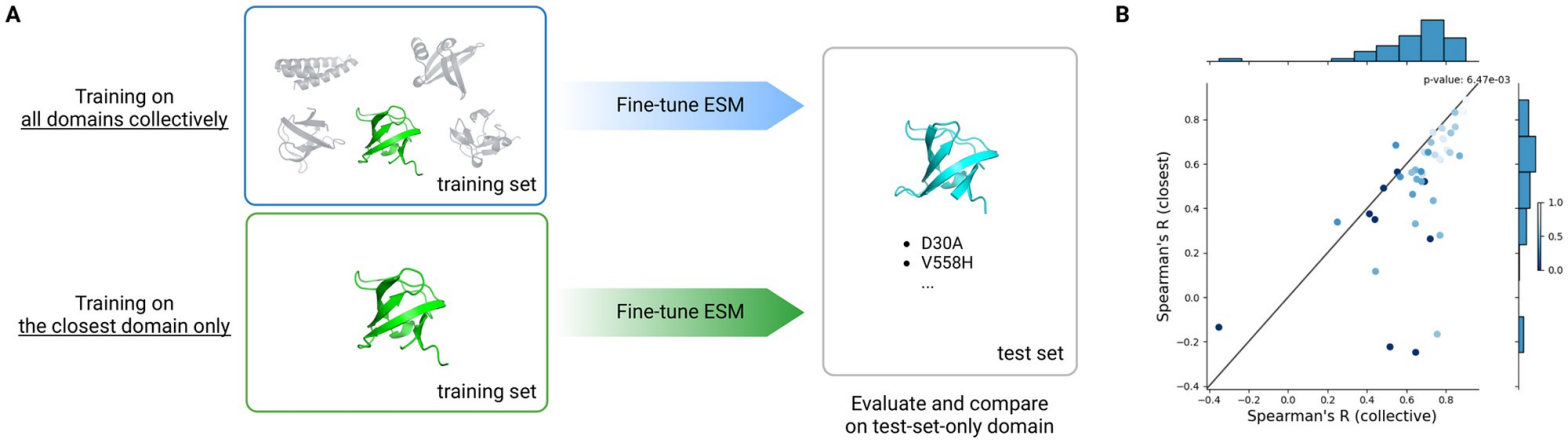
Comparison between transfer learning from the closest protein domain in training set and training on all domains collectively. (A) Schematic of the comparison. In the case of transfer learning, we match the test-set-only protein domain in cyan with the closest domain found in the training set in green. (B) Spearman’s R in the test set-only protein domains (x-axis) by learning from all domains collectively and (y-axis) by learning from the closest training-set domain alone. Samples(s) located under the diagonal line indicate better performance by learning collectively. The closest training-set domains were identified primarily by sequence alignment using MMseqs2, then by structure alignment using Foldseek, or discarded when no match was found in either case. The color bar indicates the highest sequence identity to any training-set domains and 0% was assigned when no sequence alignment was found. Statistical significance is performed with Wilcoxon’s rank sum test (p-value = 6 x 10^-3^).

Extrapolating to test-set-only domains clearly benefits from learning all domains collectively. Collective training improves Spearman’s R by 0.16 on average (p-value = 6x10^-3^), as illustrated in Fig (3). CdnL protein (PDB code: 2LQK) [40] cannot be aligned with any training sequence and instead was matched with its closest structural alignment (PDB code: 2BTT) [41] with Foldseek [42]. Collective training increased CdnL’s Spearman R from -0.25 to 0.65. Similarly, amino-terminal domain of phase 434 repressor (PDB code: 1R69) [43] was matched by structural alignment to a redesigned protein G (PDB code: 1EM7) [44] with a TM-score of 0.23, and gained 0.74 in Spearman’s R from -0.22 to 0.52. Looking into the domains with sequence alignment to the training set, WW domain from APBB3 (PDB code: 2YSC) shares 47% identity with its training-set partner (PDB code: 1WR7) and yet still benefits from multi-domain training with an improvement of 0.32. In contrast, uncharacterized yahO protein remains a difficult target. Compared to training on its closest training-set domain (PDB code: 1IGV), learning on multiple domains only improves the correlation from -0.35 to -0.14. Overall, these results highlight the benefits of a protein stability dataset on a diverse collection of protein domains for generalization to previously understudied targets.

Although the improvement brought by collective training highlights the benefits of a consistent large-scale dataset on folding stability, it is still unclear whether the improvement originates from the shared knowledge on folding stability across multiple domains or the sheer number of samples. The discrepancy in dataset size is significant as an individual domain only constitutes up to 7k sequences, less than 2% of the training set on the collection of protein domains.

In addition to extrapolating to test-set-only protein domains, we conducted a similar comparison on the impact of training on a collection of protein domains on interpolation on previously observed protein domains. Overall, performance on sequences from training-set domains is marginally uplifted by learning from a multi-domain dataset. Illustrated in S2 Fig, learning from an ensemble of protein domains weakly outperforms models trained on the same domain by an average of 0.03 (p-value = 2x10^-2^). However, the margin is slim. 72% of the domains have Spearman’s R only change by 0.1.

### Comparison with existing models on larger proteins

Although natural proteins often span between 200 and 400 residues [45], ESM_therm_ is fine-tuned on sequences no longer than 72 residues in length. To explore its performance under this limitation, we benchmarked our model on seven stability-related datasets on larger proteins and compared our results with state-of-the-art covering different methodologies in Tables 1 and 2. These include Rosetta Cartesian ΔΔG for molecular modeling, MUPro for support vector machine (SVM) on traditional sequence features, RaSP for structure-based CNN, ELASPIC-2 which employs a machine-learning model based on both structure and sequence embedding, and unsupervised prediction from ESM-2 [46].

**Table 1:** Comparison of Spearman’s R across methods on individual DMS datasets. All evaluation is restricted to point mutations, except our pLM on the mega-scale dataset. We also report unsupervised prediction from pretrained ESM-2 to contrast with supervised approaches. While the mega-scale dataset from Tsuboyama et al. covers multiple protein domains [32], all other datasets studied only one target protein.

Dataset	Length	Supervised Prediction					Unsupervised Prediction	
		Rosetta	MUPro	RaSP	ELASPIC-2	ESM_therm_	ESM-2 (35M)	ESM-2 (3B)
Mega-scale dataset	40–72	0.61	0.31	**0.64**	**0.64**	**0.65**	0.36	0.43
BglB dataset	445	-0.01	-0.02	0.02	-0.11	-0.11	-0.12	-0.12
Bgl3 dataset	510	0.49	0.10	0.44	**0.56**	0.06	0.43	0.53
Acetyltransferase dataset	177	0.33	0.16	0.30	**0.49**	0.03	0.25	**0.50**
Lipase EstA dataset	212	**0.48**	0.06	0.41	**0.47**	0.04	0.26	0.28
PTEN dataset	403	0.44	0.17	0.41	**0.47**	0.04	0.26	0.25
Methyltransferase dataset	245	0.48	0.21	0.40	**0.58**	0.03	0.42	0.46

**Table 2:** Overview of benchmarked DMS datasets on protein stability.

Dataset name	Protein name	Protein length	Measured quantity	No. of sequences
Mega-scale dataset [32]	multiple	40–72	cDNA display proteolysis	100,794 (test set)
BglB dataset [47]	Beta-glucosidase (BglB)	445	melting temperature (T_m_)	157
Bgl3 dataset [49]	Beta-glucosidase (Bgl3)	501	catalytic susceptibility to heat shock	2,999
Acetyltransferase dataset [51]	Gentamicin 3-N-acetyltransferase	177	chemical stability	1,801
Lipase EstA dataset [50]	Lipase EstA	212	melting temperature (T_50_)	2,172
PTEN dataset [52]	PTEN	403	protein abundance	5,083
Methyltransferase dataset [53]	Thiopurine S-methyltransferase	245	protein abundance	3,648

We observe comparable performance in predicting the thermostability of test-set-only protein domains across all models except MUPro. Our pLM achieves a Spearman’s R of 0.65, compared to 0.64 from RaSP and ELASPIC-2, and 0.61 from Rosetta molecular modeling. MUPro finishes last by scoring 0.31. Drawing an interesting parallel between datasets, Huang et al. reported direct melting temperature measurements of beta-glucosidase active-site mutants (PDB code: 2JIE) manually selected based on biophysical knowledge [47, 48], while Romero et al. leveraged a log-enrichment value to gauge the stability for a similar beta-glucosidase (PDB code: 1GNX) in a site-saturated fashion [49]. The former closely resembles a smaller-scale study guided by domain knowledge in contrast to the latter dataset that leverages a parallelized assay. Despite an identical alpha-beta barrel scaffold and catalytic mechanism, and a shared sequence identity 48%, most models achieve Spearman’s R above 0.4 on the Bgl3 dataset, and no method correlates with BglB dataset. This highlights the potential impact of sampling and assay through a comparative setting.

Trained specifically on small protein domains, ESM_therm_ does not generalize to other datasets on larger protein sequences. In a collection of direct [50] and indirect [51–53] stability measurements, state-of-the-art methods outperform our pLM convincingly. Cartesian ddG in Rosetta achieves generalizability through molecular modeling with a correlation between 0.33 and 0.48. Simultaneously, RaSP is built on top of Cartessian ddG and dramatically speeds up the protocol with marginal correlation setbacks. Overall, ELASPIC-2 ranks highest with a Spearman’s R of 0.42–0.58 while our pLM correlates to none of these datasets.

Another intriguing observation is the performance of unsupervised predictions from pLM. While ESM-2 is less capable of predicting stability changes within the mega-scale dataset, it excels in datasets where indirect stability measurements correlate with function. These include log2-enrichment value which characterizes how catalytic activity reacts to heat shock in Bgl3 dataset and the intracellular abundance of the protein in the acetyltransferase dataset. Conversely, ESM-2 has a comparably weaker performance for proteolysis folding stability in the mega-scale dataset and chemical stability in the Lipase EstA dataset, where the assays measure stability directly. We also highlight the impact of fine-tuning by benchmarking the unsupervised prediction from 35M-parameter ESM-2 against our fine-tuned ESM_therm_ of the same model size. Supervised prediction improves the correlation from 0.36 to 0.65.

## Discussion

Although our model generalizes reasonably well to new small protein domains in the mega-scale thermostability dataset, it is substantially weaker on larger proteins. Studies have established a strong correlation between the parallelized assay and direct measurement of thermostability [54]. However, we cannot rule out that our language model is biased towards dataset-specific details, including experiment conditions and sampling distribution of protein sequences. One hypothesis is that our pLM is biased toward shorter sequences, while geometric learning do not suffer from the same pitfall and already performs better in unsupervised prediction [55]. The protein domains on which we trained are limited to 40 to 72 amino acids in length, a stark contrast to the 177- to 501-residue-long sequences in our additional DMS benchmark. This might suggest that fine-tuned pLM stability predictors would benefit from a large-scale folding stability dataset on longer sequences.

**Fig 4:**
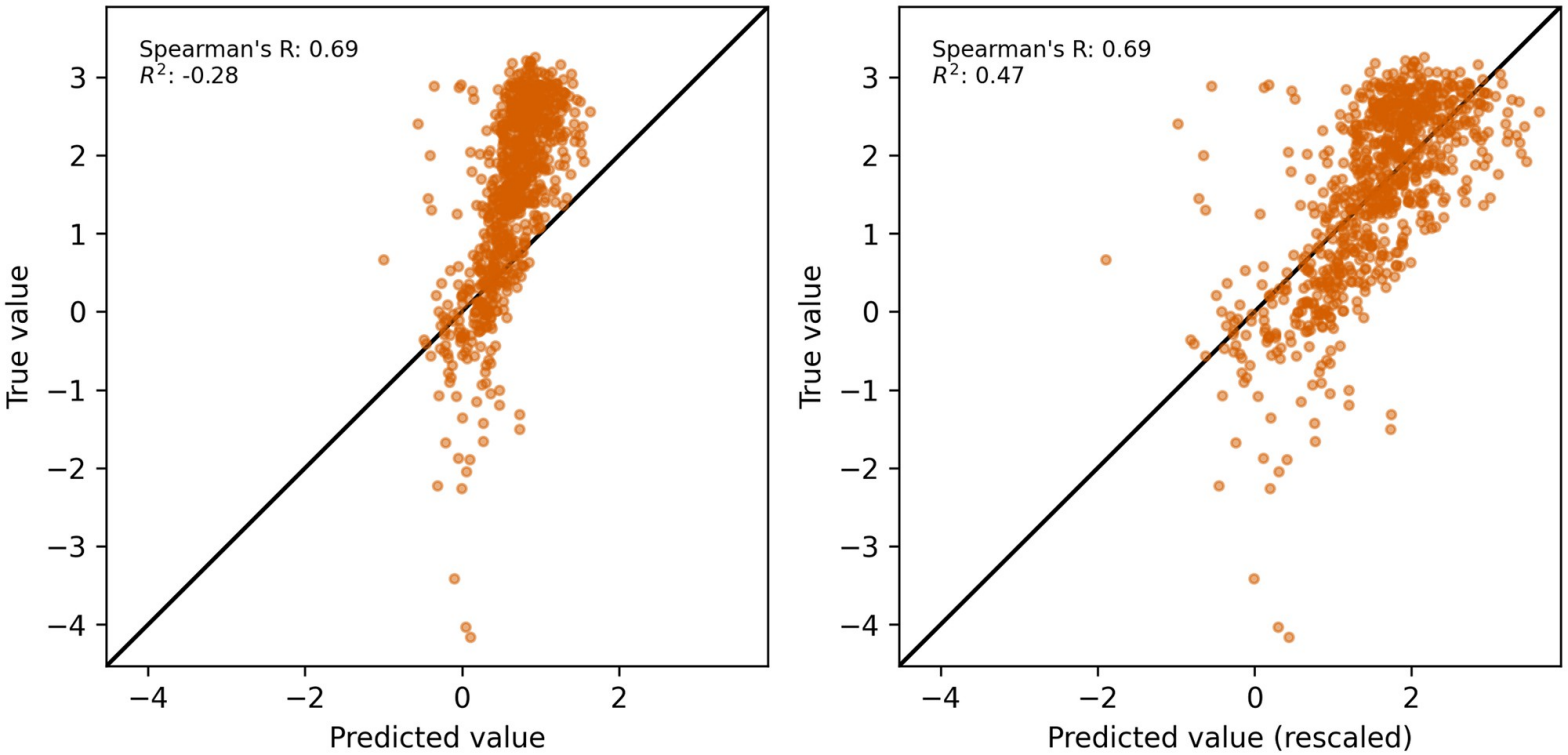
Impact of recalibration. (Left) Miscalibration between prediction and true value on the stability of DNA-binding arginine repressor. (Right) Recovered agreement between prediction and stability measurement through linear rescaling on the same set of data.

While most methods can rank ΔΔG between mutants successfully, predicting ΔG is still challenging. Our predictions often suffer from an offset and/or scale differently when compared to the experimental ΔG of the test-set-only domains (S3 Fig) and other methods might share the same problem. For example, Rosetta Cartesian ΔΔG follows a different energy unit (Rosetta Energy Unit), and it might not be suitable to be compared directly to kcal mol^-1^. However, the misalignment can be easily resolved by a simple linear regression between model prediction and experiment. Upon recalibration per protein domain, the root mean square error from our model improved from 1.34 to 0.83 and R^2^ from -0.85 to 0.45, averaged across all test-set-only domains. For instance, our model scores a negative R^2^ on DNA-binding arginine repressor before recalibration and improves to 0.47 after rescaling, while Spearman’s R remains the same at 0.69 regardless of any monotonic transformation (Fig 4).

## Conclusion

In this work, we demonstrate that folding stability prediction is possible using a protein language model. Enabled by large-scale protein stability measurements, we fine-tuned ESM-2 on the absolute folding energy of small protein domains. This approach generalizes successfully to protein domains distal from the training set, showing the potential of transfer learning to reduce experimental burden. Furthermore, our result highlights the benefits of training collectively on all protein sequences instead of mutagenesis study on a single wildtype. Although its performance on larger protein scaffolds is lagging behind state-of-the-art, a folding stability dataset of larger proteins might be vital to improving the generalizability of ESM_therm_.

## Methods and materials

### Protein language model and fine-tuning protocol

ESM-2 is a transformer pLM pre-trained on masked-language-model (MLM) objective on UniRef50. We fine-tuned the model on whole-sequence regression task with a classification head on the starting token. All parameters were trainable in fine-tuning, and used a local batch size of 128 and a global batch size of 2048. We trained the model on A100 GPU at half precision with a patience of 500 steps. We report all test-set-only evaluations on the checkpoint with the best performance on the validation set.

We performed hyperparameter selection on model size (8, 35, 150, and 650 million parameters) (S1 Table), and selected the 35-million-parameter model to balance prediction performance and compute speed. In addition, we performed an ablation study on pretraining. Model with pretraining has a superior advantage over that with random initialization (S1 Fig).

### Dataset construction

Tsuboyama et al. measured the folding stability of 1.8M measurements derived from 542 protein domains by cDNA display proteolysis [32]. We aggregated measurement(s) with the identical protein sequence, regardless of their DNA sequence(s), into a single entry. In cases where the DNA sequence was unique while sharing the same protein sequence, we evaluated the standard deviation of ΔG and log K_50_. We removed measurements when the standard deviation of ΔG was greater than 2 kcal mol^-1^ or that of log K_50_ was greater than 0.5 and we kept only domains with at least 100 measurements by protein sequence. This reduced the number of entries from 851,552 protein sequences from their original criteria (K50_dG_Dataset1_Dataset2.csv) to 527,785 protein sequences and 258 natural and 203 *de novo* protein domains.

Under the hierarchical nature of this dataset, by which multiple domains are constituted and each domain holds a collection of multiple mutants, the definition of model generalizability has two layers. The first is the ability of the model to generalize to mutants on training protein domains, and the second is that on test-set-only domains. To evaluate the model on both training and test-set-only domains, we split our dataset into train, validation, and test sets by domains as illustrated in Fig 1. 10% of all domains, defined by wildtype by the authors, are randomly drawn and all of their mutants are assigned to the test set. Mutants are randomly assigned to train-validation-test sets in an 80–10-10 ratio for the remaining domains.

### Sequence and structural alignment

We implemented sequence clustering and alignment through MMseqs2 [56]. For clustering, we clustered the domain wild-type sequences using a similar strategy in constructing the Uniclust database. We dropped prefiltering for all-to-all pairwise alignment. For Foldseek, we searched for the structural identity based on AlphaFold structures from Tsuboyama et al [32]. Unless otherwise specified, we used the default parameters in both MMseqs and Foldseek. The implementation details of alignment can be found src/esmtherm/alignment in the GitHub repository.

### Matching test-set-only domains

We fine-tuned ESM-2 (esm2_t12_35M_UR50D) on each of the 416 protein domains in the training set as our independently learned models. We first matched each test-set-only domain to its closest partner in the training set by the highest sequence identity using MMseqs2. In the case where no sequence alignment is identified, we matched test-set-only domain by the highest structural identity by Foldseek. In the case that neither is identified, the test-set-only domain was not compared. Pairwise comparisons of interpolation and extrapolation are performed in Wilcoxon’s rank sum test.

### Benchmark protein dataset selection

Given the intensive computing resource required to benchmark Rosetta, we limited ourselves to six DMS datasets on direct and indirect stability measurements from ProteinGym [57, 58], and another independent mutational dataset (BglB) from Huang et al. [47] to cover a range of assays. Nutschel et al. reported the thermostability (ΔT_50_) of *Bacillus subtilis* Lipase A [50], whereas Dandage et al. reports chemical stability on Gentamicin 3-N-acetyltransferase [51]. Contrary to direct stability measurements, PTEN and Methyltransferase datasets correlate with stability through enhancement or depreciation of intracellular abundance as an indirect indicator [52, 53]. The pair of Bglb and Bgl3 datasets was chosen for a comparative study on the impact of sampling and measurement assays. Bgl3 from Romero et al. and BglB datasets [47, 49] share homologous beta-glucosidase sequences but differ in log enrichment value and melting temperature (T_m_) as indirect and direct thermostability measurements.

## Supporting information

S1 Fig Ablation of pretraining measured in Spearman’s R. Each sample is a collection of mutants from a test-set-only domain. The x-axis is Spearman’s R of a test-set-only domain with pretraining. The y-axis is that from randomly initialized model. The color bar on the right represents the closest sequence identity in the train and validation set domains. The statistical assessment was performed using Wilcoxon’s rank sum test.
(TIF)

S2 Fig Comparison between learning from the same protein domain only and training on all domains collectively. (A) Schematic of the comparison. (B) Spearman’s R on test mutants whose protein domains are also present in the training set. The x-axis represents learning from all domains collectively and the y-axis is learning from the same protein domain alone. Domain(s) located under the diagonal line indicate better performance when learning collectively. Statistical significance is performed with Wilcoxon’s rank sum test.
(TIF)

S3 Fig Offset in ΔG prediction on wildtype sequences. The x-axis is the ΔG prediction from ESM_therm_ and the y-axis is the experimental ΔG label. The Spearman’s R across all wildtypes in test-set-only protein domains is 0.39.
(TIF)

S1 Table Performance evaluation on different model sizes on test set. Metrics are evaluated on each individual domain, and then aggregated into mean and standard deviation over all domains. All models have similar performance metrics with esm2_t12_35M_UR50D except esm2_t6_8M_UR50D on Spearman’s R (p-value < 5x10^-2^).
(PDF)

S1 Spreadsheet Performance evaluation per protein domain. (CSV)

S2 Spreadsheet Model prediction per protein sequence. (CSV)
