# Supplementary figures and images for: Protein stability prediction by fine-tuning a protein language model on a mega-scale dataset
Source: PLoS Comput Biol. 2024 Jul 22;20(7):e1012248. doi: 10.1371/journal.pcbi.1012248 (PMC11293664; doi:10.1371/journal.pcbi.1012248)

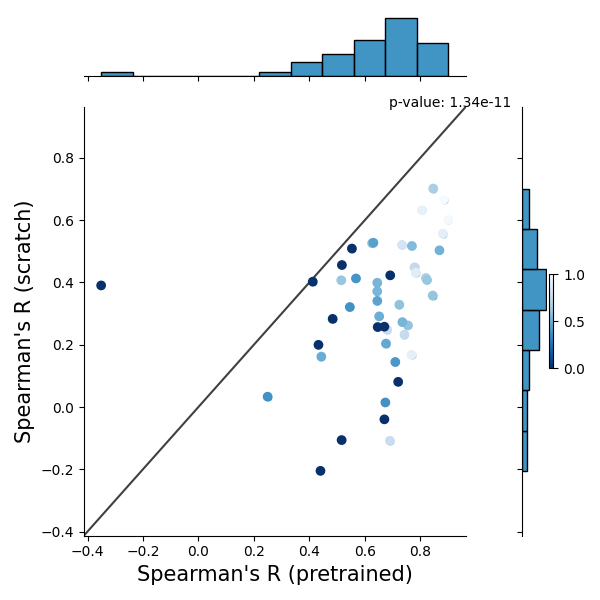

Supplement: S1 Fig — Each sample is a collection of mutants from a test-set-only domain. The x-axis is Spearman’s R of a test-set-only domain with pretraining. The y-axis is that from randomly initialized model. The color bar on the right represents the closest sequence identity in the train and validation set domains. The statistical assessment was performed using Wilcoxon’s rank sum test. (TIF) [file pcbi.1012248.s001.tif]

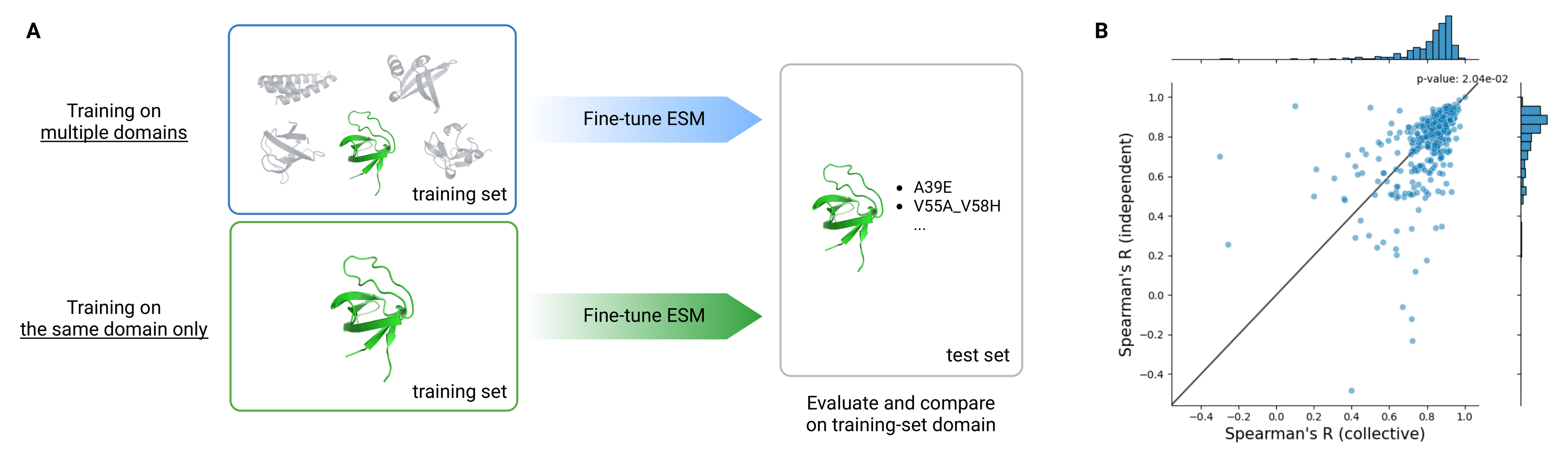

Supplement: S2 Fig — (A) Schematic of the comparison. (B) Spearman’s R on test mutants whose protein domains are also present in the training set. The x-axis represents learning from all domains collectively and the y-axis is learning from the same protein domain alone. Domain(s) located under the diagonal line indicate better performance when learning collectively. Statistical significance is performed with Wilcoxon’s rank sum test. (TIF) [file pcbi.1012248.s002.tif]

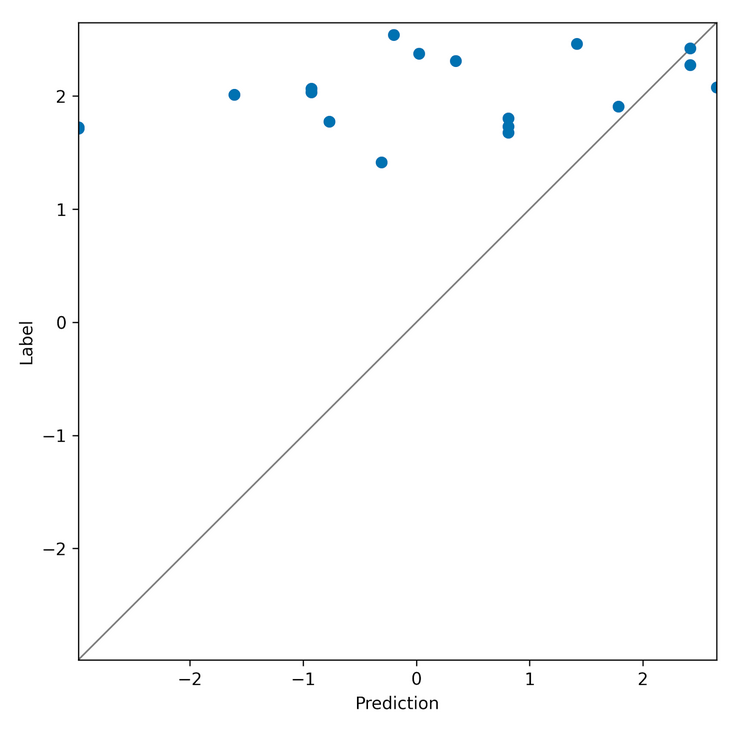

Supplement: S3 Fig — The x-axis is the ΔG prediction from ESMtherm and the y-axis is the experimental ΔG label. The Spearman’s R across all wildtypes in test-set-only protein domains is 0.39. (TIF) [file pcbi.1012248.s003.tif]
