# Supplementary material for: Protein stability prediction by fine-tuning a protein language model on a mega-scale dataset
Source: PLoS Comput Biol. 2024 Jul 22;20(7):e1012248. doi: 10.1371/journal.pcbi.1012248 (PMC11293664; doi:10.1371/journal.pcbi.1012248)
Supplement: S1 Table — Metrics are evaluated on each individual domain, and then aggregated into mean and standard deviation over all domains. All models have similar performance metrics with esm2_t12_35M_UR50D except esm2_t6_8M_UR50D on Spearman’s R (p-value < 5x10^-2^). (PDF) [file pcbi.1012248.s004.pdf]

Table S1: **Performance evaluation on different model sizes on test set.** Metrics are evaluated on each individual domain, and then aggregated into mean and standard deviation over all domains. All models have similar performance metrics with esm2_t12_35M_UR50D except esm2_t6_8M_UR50D on Spearman's R (p-value $< 5x10^{-2}$).

Model name	MSE	Spearman's R	R^2
esm2_t6_8M_UR50D	2.48±3.14	0.57±0.21	0.14±0.23
esm2_t12_35M_UR50D	2.10±2.52	0.65±0.21	0.18±0.26
esm2_t30_150M_UR50D	2.02±2.28	0.70±0.15	0.15±0.24
esm2_t33_650M_UR50D	1.91±2.05	0.72±0.12	0.17±0.26
